# Targeting Remyelination in Spinal Cord Injury: Insights and Emerging Therapeutic Strategies

**DOI:** 10.1111/cns.70193

**Published:** 2024-12-26

**Authors:** Abdullah Al Mamun, Zhou Quan, Peiwu Geng, Shuanghu Wang, Chuxiao Shao, Jian Xiao

**Affiliations:** ^1^ Central Laboratory of The Lishui Hospital of Wenzhou Medical University, The First Affiliated Hospital of Lishui University Lishui People's Hospital Lishui Zhejiang China; ^2^ Oujiang Laboratory (Zhejiang Lab for Regenerative Medicine, Vision and Brain Health), School of Pharmaceutical Sciences Wenzhou Medical University Wenzhou Zhejiang China; ^3^ Department of Wound Healing The First Affiliated Hospital of Wenzhou Medical University Wenzhou Zhejiang China

**Keywords:** neurological impairments, regeneration, remyelination, spinal cord injury

## Abstract

**Introduction:**

Spinal cord injury (SCI) is a severe neurological disease characterized by significant motor, sensory, and autonomic dysfunctions. SCI is a major global disability cause, often resulting in long‐term neurological impairments due to the impeded regeneration and remyelination of axons. A SCI interferes with communication between the brain and the spinal cord networks that control neurological functions. Recent advancements in understanding the molecular and cellular mechanisms of remyelination have opened novel therapeutic interventions.

**Method:**

This review systematically sourced articles related to spinal chord injury, remyelination, regeneration and pathophysiology from major medical databases, including Scopus, PubMed, and Web of Science.

**Results:**

This review discusses the efficacy of targeted therapy in enhancing myelin repair after SCI by identifying key molecules and signaling pathways. This explores the effectiveness of specific pharmacological agents and biological factors in promoting oligodendrocyte precursor cell proliferation, differentiation, and myelin sheath formation using in vitro and in vivo models. Targeted therapies have shown promising results in improving remyelination, providing hope for functional recovery in SCI patients.

**Conclusions:**

This review demonstrates challenges and future perspectives in translating findings into clinical practice, emphasizing safety profiles, delivery method optimization, and combinatory therapy potential. This review also supports the possibility of targeted remyelination therapies as a promising strategy for SCI treatment, paving the way for future clinical applications.

## Graphical abstract

Exploring the potential targeted therapy of remyelination after spinal cord injury: The review demonstrates the possibility of stimulating oligodendrocyte precursor cell proliferation, differentiation, and myelin sheath formation in both in vitro and in vivo SCI models through molecular pathway modulation and pharmacological agents. Targeted therapies have shown promising results in improving remyelination, providing hope for functional recovery in SCI patients.

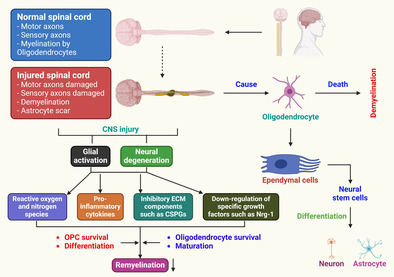

## 1 Introduction

A severe neurological disease that results in physical dependence, morbidity, psychological distress, and financial burden is spinal cord injury (SCI). Its worldwide prevalence has risen from 236 to 1298 instances per million inhabitants within 30 years. An estimated 250,000 to 500,000 people worldwide are affected by SCI [1]. Every patient with SCI must pay more than $3 million in lifetime expenses, and Canada's estimated yearly economic burden is close to $2.67 billion [2]. Researchers focus on enhancing neural regeneration in SCIs and remyelination, using biomaterials to protect oligodendrocytes and improve brain function [3]. SCI is a severe disease with high disability rates. Researchers have attempted to understand its pathological mechanisms and identify strategies for promoting axon regeneration and neural circuit remodeling. Bioactive materials and stem cell advancements focus on forming intermediate neural networks for neural regeneration [4]. SCI can be divided into primary and secondary mechanisms, with primary injury determining a neurological ability [5]. Demyelination after SCI is caused by extended and distributed oligodendrocyte cell death. Numerous researchers have used myelin‐producing cell transplants to treat contusion SCI and restore the missing myelin. Cellular transplantation restores function after injury, with functional recovery linked to the extent of myelin regeneration. Cellular transplantation is a valuable treatment for SCI, and evaluating the clinical value of remyelination after SCI is essential. Long‐term demyelination increases axonal susceptibility to degeneration, contributing to long‐term functional deficits linked to SCI. Cellular transplantation is the main method used to increase remyelination after SCI. This discusses strategies and new, unproven therapeutic approaches to regenerate myelin by differentiating nearby endogenous cells [6]. Recent advancements in understanding oligodendroglia biology, propagation, migration, differentiation, maturation, and myelination have sparked interest in remyelination as a potential restoration strategy for SCI [7]. Myelin integrity is crucial for CNS physiology and functional restoration after SCI. Therapeutic approaches include genetic manipulation, immunomodulation, glial scar manipulation, and cell transplantation to restore oligodendrocytes and myelin [8]. Axons can grow through glial scarring and Nogo‐containing white matter or resist growth without injury or stimulation. Unchecked expansion in the CNS can be disruptive and detrimental to functionality. According to Liu et al. [9], the corticospinal tract in mice can grow massively when the phosphatase and tensin homolog (PTEN) gene is removed. The optic nerve can regenerate when PTEN expression is blocked [10], as this activates the phosphokinase B and mammalian target of the rapamycin (AKT/mTOR) pathway [11]. Spinal cord adult progenitor cells differentiate into new oligodendrocytes (OLs), remyelinating spared axons. The new OLs can be generated up to three months after injury, unsheathed axons, and colocalized with MBP [12]. A multitherapy using nanomedicines with triiodothyronine, ibuprofen, and mNGF improves myelin repair in patients with irreversible SCI, demonstrating short‐term anti‐inflammatory effects and long‐term improvements in myelination [13]. The endogenous repair of SCIs in mice reveals the generation of new OLs and myelin. The chronic demyelination, including nodal protein spreading and Nav1.2 upregulation, could trigger long‐term remyelination, potentially enhancing post‐SCI myelin repair through oligodendrocyte precursor cell (OPC) processes [14]. Neural precursor cells (NPCs) enhance functional recovery from SCI in rats, suggesting remyelination as an essential therapeutic target [15]. To ensure the inclusion of the most appropriate articles in this review, an in‐depth search was carried out on prominent medical, biological, and chemical databases, including Scopus, PubMed, and Web of Science. The search used the keywords listed below: “remyelination, SCI, and therapeutic strategies.” Furthermore, the secondary keywords used were “pathophysiology, spinal cord regeneration, and drug delivery systems.” The review highlights the potential for targeted therapy to improve remyelination after SCI. Novel interventions have significantly improved neural repair and functional recovery, providing valuable insights for future research and therapeutic strategies in managing SCI.

## 2 Pathophysiology of SCI

**FIGURE 1:**
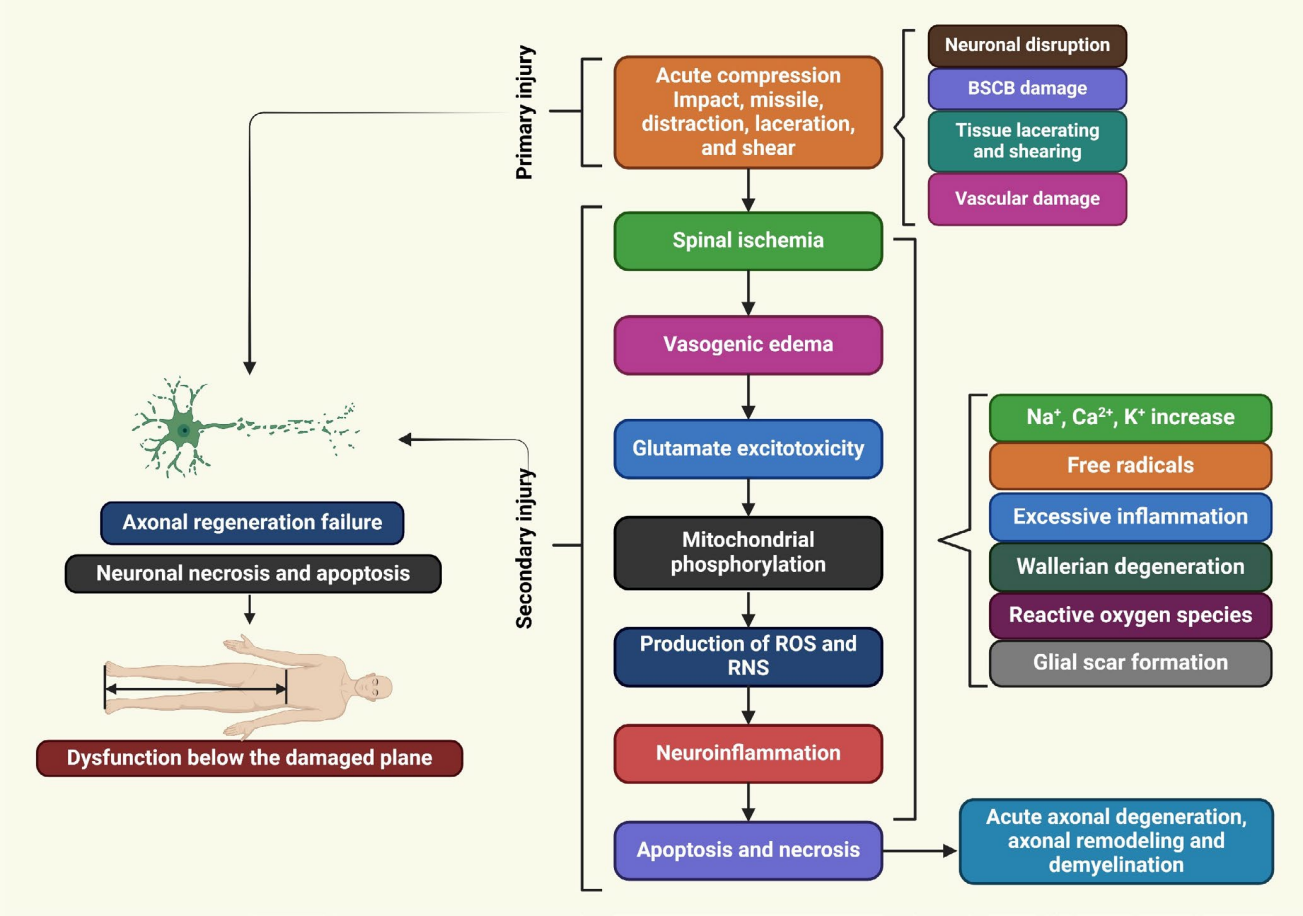
The pathophysiology of SCI leads to sensory, motor, and autonomic dysfunction, resulting in paralysis or altered sensation. Primary and secondary injury mechanisms contribute to tissue damage and neurological deficits, complicating both treatment and recovery processes.

SCI is a complex disease that results in no return in a patient's ability to repair or regenerate their function. Recent investigations showed the intricate mechanisms underlying SCI, prompting the development of new treatment methods. SCI pathophysiology, anatomy, and molecular approaches for neuroprotection or neuroregeneration. An effective treatment can slow the progression of secondary SCI damage and promote functional recovery by focusing on various pathologic processes [16]. SCI is a traumatic condition with two stages: primary damage and secondary damage. Primary damage is caused by local dysfunction, while secondary damage is determined by systemic and cellular factors [17]. Post‐injury, spinal cord vascular supply disruption, hypotension, hypovolemia, neurogenic shock, and bradycardia are common clinical presentations triggered by severe bleeding and neurogenic shock. Vasospasm is when immune cell extravasations near an injury site press on damaged spinal tissues, thereby reducing blood flow [18]. Spinal cord ischemia causes ionic, vasogenic, and cytotoxic oedema, while healthy bodies maintain a healthy balance through passive Cl^−^inflow via chloride channels and Na^+^ influx through aquaporin water channels. A pathological state disrupts the equilibrium between solute and water inflow in the intracellular compartment, causing cell edema, cytoskeletal loss, and cell death [19]. Ionic oedema is characterized by increased blood‐spinal cord barrier permeability, leading to the loss of ions and water due to trans‐endothelial ion transport. Vasogenic oedema is caused by endothelial damage and inflammation, causing pore size expansion and allowing large plasma molecules to cross the cell membrane [20]. Acute secondary damage persists 48 h after onset, causing prolonged bleeding, swelling, and inflammation, leading to significant necrosis and elevated levels of inflammatory and structural indicators in the cerebrospinal fluid (CSF) [21]. SCI is a complex pathophysiology (Figure 1) involving primary and secondary injuries, mechanical damage, and immune, nervous, and vascular system issues [22, 23]. A spinal cord hemisection injury in nonhuman primates demonstrated activated macroglia and microglia, no reactive astrocytic responses, and a thick fibrotic scar near the epicenter [24]. The temporal changes in spinal cords damaged by injury demonstrated significant dynamic alterations three days post‐injury, including a second wave of microglial activation [25]. Spinal ischemia, vasogenic edema, glutamate excitotoxicity, neuroinflammatory factors, mitochondrial phosphorylation, nitric oxide synthase (NOS) production, and chronic stages involve axon degeneration, remodeling, and glial scar formation [26]. Glutamate interacts with metabotropic and ionotropic receptors, leading to increased concentrations during SCI. This causes long‐term excitotoxicity and cell death [20]. Abnormal glutamate excitation can be caused by various factors such as mechanical stress, cell development, Na^+^/K^+^ ATPase failure, lipid peroxidation, and 4‐hydroxynonenal synthesis [27]. Apoptosis and necrotic cell death are further increased by the hyper‐activation of NMDA and AMPA receptors, which increases the influx of Ca^2+^ and Na^+^ ions [20]. Elevated glutamate in necrotic cells increases Na^+^ and Ca^2+^ concentrations, causing ionic flow disruptions. This leads to mitochondrial respiration, energy depletion, and axonal membrane depolarization, causing cytotoxic oedema, axonal acidosis, and mitochondrial dysfunction [19, 20].

## 3 Spinal Cord Regeneration

New methods for enhancing neuroplasticity and axon regeneration post‐SCI involve neuronal activity control, neural stem cell transplants, intrinsic signaling, and neuronal extrinsic environment [28]. The spinal cord suffered severe damage, with thousands of axons entering the contusion site. Hill et al. [29] evaluated the origins of the fibers that developed into the contusion site. The bulbs, destroyed by the contusion, remained visible in the CST for one to eight months after the injury. The lesion matrix was filled with numerous CST axons, with reticulospinal fibers observed at three months and detailed at subsequent stages. The axons penetrated the contusion site's gliotic tissues, degrading white matter to reach the damaged site. The hyaluronate‐binding protein (GHAP) expression was divided into permissive and nonpermissive [30]. All cultured astrocytes stimulated neurite outgrowth from various embryonic CNS neurons, regardless of lineage, shape, immunologic type, or differentiation agent treatment [31, 32]. Reactive astrogliosis inhibits axonal regrowth in vivo [33]. Spinal cord contusion injuries cause extreme reactive astrogliosis, where glial cells from injured cords inhibit new axon growth [34]. A study found a robust fibrotic scar in the hemisected monkey spinal cord, indicating primate and possibly human SCI differs from rodent SCI [24]. Profibrotic and angiogenic connective tissue growth factors inhibit axon growth [35]. Optic axons can easily pass through regions with dense glial scars, and reactive astrocytes in the mediobasal hypothalamus aid in axonal regeneration [36]. The medial cholinergic route in the brain regenerates and restores laminar patterns through the development of “glial scars” [37]. Permissive autologous BMSCs were grafted into adult rats' mid‐cervical SCI sites, demonstrating thick NG2 deposits and widespread astrocytosis [38]. Studies show spinal tract regeneration without Nogo, glial scars, or CSPG. Cell transplants, cAMP, neurotrophins, the PTEN/AKT/mTOR pathway, and injury‐inactivated regenerative mechanisms stimulate neural growth and proliferation [39].

## 4 Mechanism of Regeneration After SCI

**FIGURE 2:**
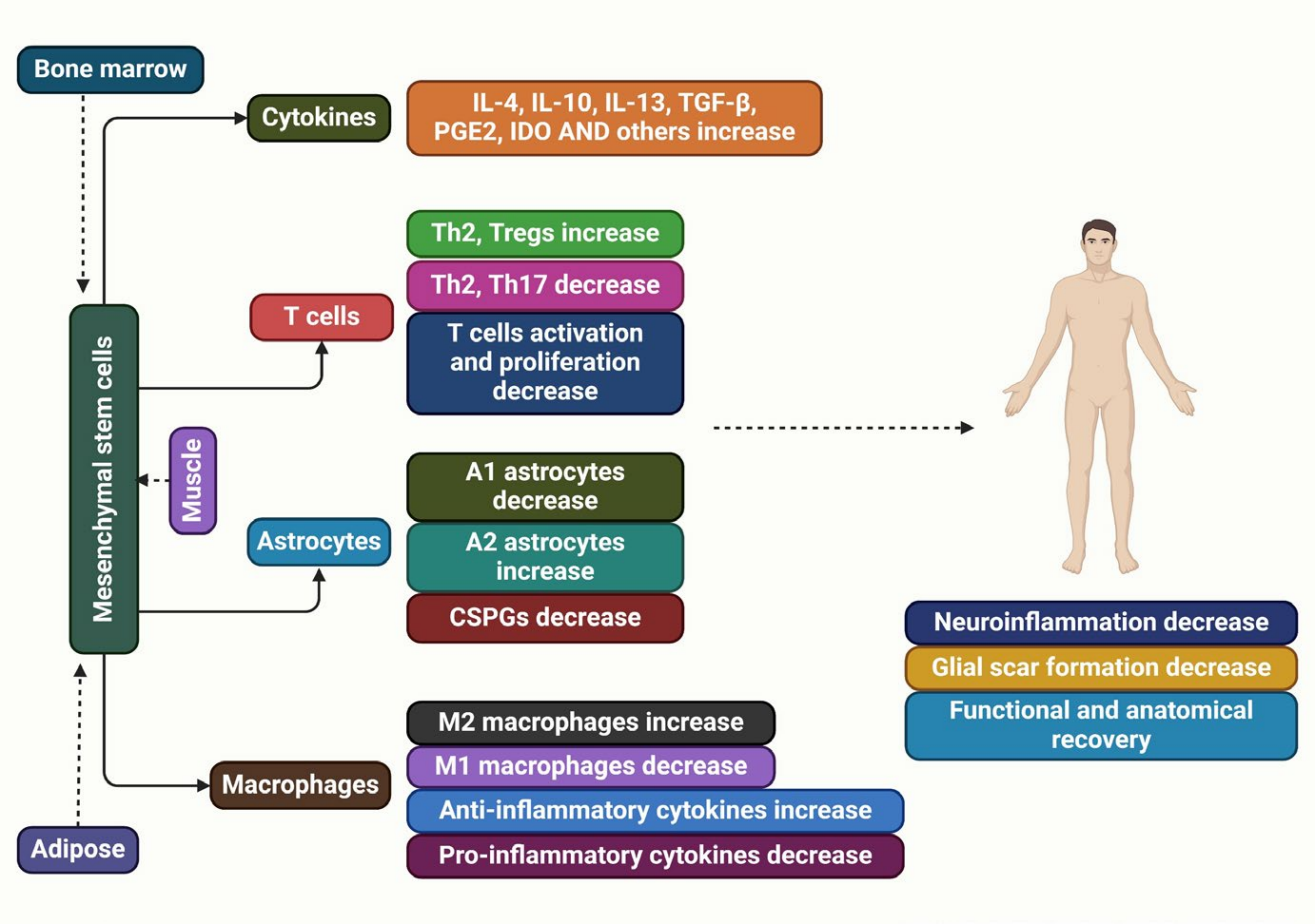
Mesenchymal stem cell transplantation aids functional and anatomical recovery in SCI patients by reducing inflammation and glial scar formation.

Understanding the cellular and molecular mechanisms inhibiting regeneration and neuroplasticity in SCI is crucial for developing effective therapies, such as CSPG‐targeting strategies, to enhance functional recovery and axonal regeneration [40]. Two OMgp animals did not show significant corticospinal axon regrowth after spinal cord damage [41, 42]. A study found increased dorsal column sensory and serotonergic axon growth in OMgp mutant mice, but it's unclear if this growth is due to sprouting or regeneration. Anti‐Nogo antibody accelerates axonal regeneration and promotes behavioral recovery [43, 44]. The lack of strong regeneration in Nogo mutant lines may be due to the production of Nogo‐C, which contains the Nogo‐66 inhibitory domain [45]. The Nogo‐A, B gene trap mutant, and Nogo null mutant were reanalyzed, but the strong axon regeneration previously reported was not replicated [45, 46]. Another study on Nogo mutant mice, MAG, and OMgp mutant animals suggests that blocking all three chemicals is necessary for axon regrowth [42, 47]. The Nogo and OMgp mutations in the two triple knockouts differ, but both have the identical MAG mutation [48]. Nogo‐A,B gene trap mutant, which contains Strittmatter's Nogo mutant allele, allows for the expression of a 309 amino acid N‐terminal fragment of Nogo‐A [46]. Our Nogo mutant allele is the fully viable Nogo null mutant, which lacks the expression of all known Nogo isoforms [45]. The OMgp mutant allele found in Strittmatter is a variant of the OMgp mutant originally reported by [41], in which the starting ATG codon is replaced with a fusion eGFP‐Neo reporter/selection cassette. The study utilized a modified OMgp mutant, excluding the reporter or selection case, to assess its impact on NF1 gene expression, contrasting with Strittmatter's triple mutant [47]. Neurons can degenerate to embryonic states post‐SCI, which can be sustained for regeneration through neural stem cell transplantation [49]. Genetically modified mesenchymal stem cells facilitate axonal regeneration and protect against hypersensitivity after SCI (Figure 2) [50]. The peripheral nerve regeneration after axotomy is influenced by various internal and external factors, including activation of signaling networks, protein expression changes, and potential environmental changes [51].

## 5 Inhibitors of Myelin Linked with Synaptic Plasticity

Myelin‐associated inhibitors (MAIs) and extracellular matrix‐associated inhibitors (EMAIs) limit neurologic recovery and anatomical growth in adult CNS injury models. MAIs limit adult CNS growth, regeneration, sprouting, and plasticity, blurring the line between injury and basic plasticity studies [52]. Studies on ocular dominance plasticity highlight the physiological roles of NgR1 and PirB. Research shows that NgR1 knockout mice exhibit sustained or enhanced ocular dominance into adulthood [53], while PirB knockout mice demonstrate similar effects [54]. Two receptors, chondroitin sulfate proteoglycans (CSPGs), may limit experience‐dependent plasticity in adult CNS injury, thereby limiting ocular dominance plasticity [55]. CSPGs and myelin inhibitor systems share similarities, with axon sprouting being a key method for reducing CSPGs' effects and enhancing functional recovery [56, 57]. After SCI, axon regeneration is not significantly affected by the genetic deletion of PTPσ, one CSPG receptor [58]. Nogo and OMgp may play a role in synaptic plasticity, as neutralizing them pharmacologically or genetically increases long‐term potentiation without altering basal synaptic transmission [59, 60] and attenuates long‐term depression [61]. In a NgR1‐dependent way, Nogo‐66 or OMgp reduces LTP when applied to acute hippocampal slices [61]. Deletion of NgR1 reverses synaptic strength, suggesting myelin inhibition may impact injury‐induced axonal development and activity‐dependent synaptic plasticity, alongside NgR1 and PirB's roles in ocular dominance plasticity [53, 54]. Research on NgR1's impact on synaptic strength suggests that loss of NgR1 may enhance synaptic strength, potentially aiding behavioral recovery by altering neuronal circuitry or learning [62]. MAIs, a group of proteins on oligodendroglia membranes, are potent inhibitors that inhibit neurite outgrowth and improve functional recovery in CNS injury models. Interfering with these proteins can promote neurite outgrowth and improve recovery. This explores MAIs and potential candidates for CNS injury treatment [63]. MAIs like Nogo, MAG, and OMgp inhibit axonal growth after CNS injury, but promoting sprouting as a repair mechanism may be a more achievable therapeutic goal [64]. Spinal cord lesions limit adult neurologic recovery and growth. MAIs and EMAIs limit functional recovery, morphological rearrangements, growth, and activity‐dependent plasticity, making it difficult to differentiate between injuries and basic forms of plasticity [52]. MAIs effectively inhibit neurite outgrowth and enhance functional recovery in damaged neurons. Interfering with these proteins can benefit CNS damage models [63].

## 6 Mechanism‐Based Therapy Target of Remyelination After SCI

Remyelination is essential for axon functional recovery after SCI. Glial cells, including oligodendrocyte precursor cells, play a role in remyelination. Microglia control the inflammatory response, while astrocytes influence oligodendrocyte precursor cell (OPC) growth and differentiation [65]. Although there is little doubt that remyelination happens after SCI in humans [66, 67], there is significant disagreement regarding the extent of remyelination after SCI in humans. The presence of demyelinated axons in the spinal cord of chronically injured individuals presents a promising therapeutic target [68, 69]. Chronically demyelinated axons are frequently located close to the injury site and are usually detected utilizing spinal cross‐sections. After injury, proximal axons die away from the injury site but can continue nearby with no function. Two studies have found that the myelination condition of spared axons, which avoid lesion location, is irrelevant to function despite their frequent myelination. Three months after SCI, those authors observed no enduring demyelination in mice [70] or rats [71] with contusive injuries. Based on these studies, it can be said that following a rodent contusion injury, remyelination of the descending spinal cord pathways is complete [70, 71]. Adult brain‐derived NPCs can be transplanted into spinal cords post‐injury to repair myelin loss caused by SCI, enhancing functional recovery [72]. Cell transplants have been used to restore missing myelin, and functional recovery is linked to myelin regeneration [73, 74]. Clinical studies on cellular transplantation for SCI treatment are ongoing, with remyelination for potential benefits, as long‐term demyelination increases axonal degeneration and functional deficits [6].

### 6.1 Remyelination Protects Axons from Degeneration

Remyelination offers numerous benefits, but its connection to improved axon sparing is weak, as demyelination increases axon susceptibility [75, 76]. The link between remyelination efficiency and axonal degeneration is uncertain, with potential factors like gliosis, immunological changes, and increased sensitivity to hazardous substances [77]. Neuroprotective strategies in animal models of demyelination involve facilitating remyelination by transplanting myelinating cells [78]. A study found progressive myelin vacuolation and disease development in adult oligodendrocyte death, indicating inefficient removal of myelin debris, impaired remyelination, and compromised axonal integrity [79]. Another study demonstrated the link between delayed remyelination and axonal degeneration, showing that X‐irradiation prevents remyelination, thereby increasing axonal degeneration [80]. Swiss Jim Lambert mice infected with Theiler's virus showed remyelination, an antibody that protects axons and preserves transport function. Treatment with rHIgM22 increased remyelination, potentially preventing chronic disease impairments [81].

### 6.2 After SCI, White Matter Sparing Improves by Remyelination Acceleration

SCI can lead to ongoing axonal injury, possibly resulting in secondary damage from prolonged oligodendrocyte apoptosis, potentially lasting weeks [82]. Uncovered axons are more susceptible to damage, making remyelination faster if axons are continuously damaged. However, the speed of remyelination for improved functionality is uncertain [66]. New myelin forms six days after injury, with regenerated sheaths visible 7 days after demyelinating injections. Remyelination typically takes 10–20 days, with faster remyelination in mice [83, 84, 85]. Myelination is enhanced during pregnancy, potentially due to the hormone prolactin. Pregnant mice show increased myelin‐forming oligodendrocytes and myelinated axons, suggesting maternal white matter plasticity can repair demyelination. Prolactin acts as a potential therapeutic agent [86]. A study found that myelin regulatory factor expression in PDGFRα‐positive oligodendrocyte progenitor cells is essential for myelin regeneration after SCI [87]. Rats with SCI received media, dead NPCs, shiverer NPCs, or wild‐type mouse NPCs as transplants. Results showed trophin infusion protected gray matter, white matter, and oligodendrocytes [15].

## 7 Strategies for Enhancing Remyelination

Cellular transplantation and endogenous healing can improve remyelination in SCI. Cellular transplantation is challenging due to logistical and ethical issues. Increasing endogenous OPC recruitment and differentiation may speed up remyelination. Animal models can provide insights into these therapies (Table 1).

**TABLE 1:** An overview of in vivo research employing oligodendrocyte‐protective and remyelination molecular therapies in animal models of SCI.

Molecular therapies	Animal model	Utilization	Ref.
*Oligodendrocyte‐protective molecular therapies*			
Guanosine	Compression is used to treat contusion injuries in adult female Wistar rats	An intraperitoneal injection every day (8 mg/kg) for two weeks, beginning four hours after the injury	[88]
Selenium	Wistar rats that are adults complete corticospinal cord tract transaction	Delivered immediately after damage at varying doses of 0, 10, 30, and 50 ng/kg; injected directly into the injured site	[89]
Methylprednisolone	Female Long‐Evans rats with contusion injuries (12.5 g/cm)	15 min after the injury, 30 mg/kg; intravenous injection	[90, 91]
Rolipram	Sprague–Dawley female rats with 180 kilodynes of contusion	Every day for up to three days (0.5 mg/kg); administered by subcutaneous pump	[92, 93]
Leukemia inhibitory factor	Adult female C57Bl/6 wild‐type mice; hemispheric over section	Intraperitoneal injection administered 2.8 and 24 h after damage; daily dose (25 mg/kg) for two weeks beginning two hours after injury	[94, 95, 96]
Caspase inhibitors	Rats: Injuries from contusion (12.5 g/cm)	Intraperitoneal injection of 5 mg/kg given 4, 72, and 144 h after injury; administered for 8 days	[97, 98]
Minocycline	Adult Sprague–Dawley rats with contusion injuries (25 g/cm)	Twice daily for three days (45 mg/kg); intraperitoneal injection beginning two hours after injury (90 mg/kg)	[99]
*Remyelination molecular therapies*			
Guanosine	Contusion damage in adult female Wistar rats	8 mg/kg per day for the first 35 to 41 days following injury via intraperitoneal injection	[100]
NT‐3	Contusion damage in adult female Fisher rats	Genetically altered fibroblasts	[101]
Progesterone	Sprague–Dawley male adults; transaction injury	Four 4 mg/kg injections were given 1, 24, 48, and 72 h following the injury	[102]
BDNF	Contusion damage in adult female Fisher rats	Genetically altered fibroblasts	[101]

### 7.1 Extracellular Inhibitors of Remyelination: A Target for OPC Differentiation and Remyelination Following SCI

The lack of improvement observed after mouse PDGF‐responsive neural precursor transplantation suggests that the damage is involved in remyelination regulation [103]. Remyelination is inhibited by myelin itself. Myelin significantly reduces the expression of myelin markers in OPCs and hinders remyelination after demyelination [104, 105]. Myelin debris has been seen surrounding demyelinated axons on occasion. The study suggests that insufficient clearance of myelin debris may lead to impaired CNS remyelination, as reduced circulating monocytes weaken myelin phagocytosis and remyelination in animals [106, 107]. Heterochronic parabiosis, a surgical procedure that combines a young and an older animal to share a single circulatory system, can potentially restore remyelination in an aged mouse. In elderly animals, heterochronic parabiosis is linked to faster oligodendrocyte differentiation and remyelination [108]. Rejuvenated remyelination increases the phagocytosis rate, but if compromised, improvement decreases. Remyelination is rate‐limiting, removing myelin debris, but myelin debris remains in rodents for eight weeks post‐susceptibility [109, 110]. Lysolecithin injection induces rapid myelin clearance, causing remyelination in lysolecithin‐induced lesions. Myelin clearance is key to remyelination, with monocyte/macrophage density at lesion sites and border [104, 111, 112]. The lesion border is expected to have the highest oligodendrocyte production due to myelin debris inhibiting mature oligodendrocyte production in culture, indicating increased oligodendrogenesis [113].

### 7.2 Improvement of Remyelination With Cellular Transplantation

**FIGURE 3:**
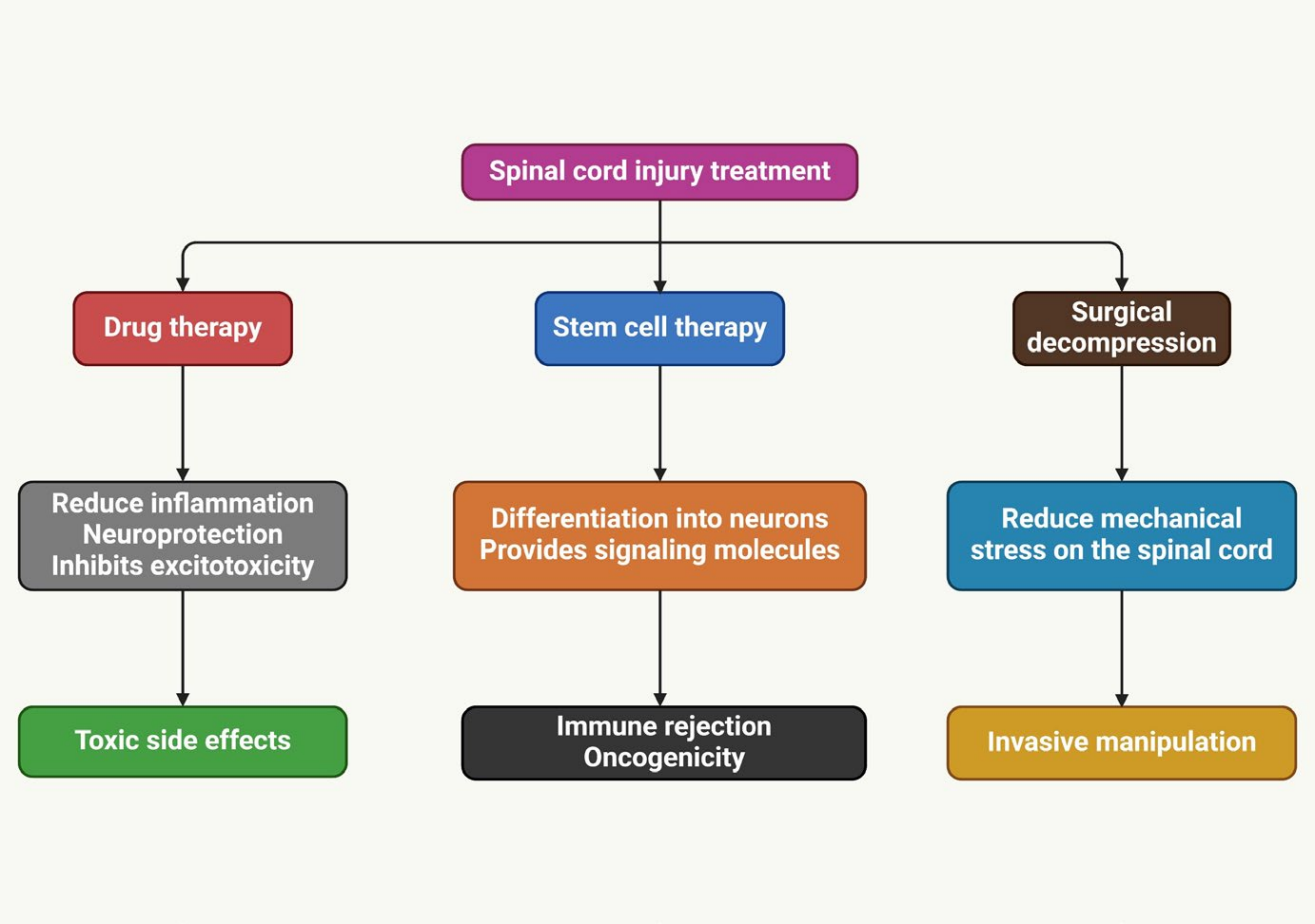
The function and limitations of current SCI treatments. Current treatments for SCI, including surgical interventions, pharmacotherapy, etc., have limitations, including incomplete function restoration, complications, and high costs.

Cellular transplantation can replace oligodendrocytes post‐trauma, demyelinating disease, or developmental abnormalities, promoting remyelination and functional recovery of SCI [77, 114, 115, 116]. Schwann cells typically remyelinate in axons near or surrounding the lesion site after exhibiting restricted migration and integration into the spared host parenchyma. In addition to Schwann cell transplantation, remyelination can lead to positive outcomes, such as increased axonal development at the lesion site [117, 118, 119]. SCI treatment (Figure 3) improved functional recovery, increasing white matter sparing and remyelination of host axons post‐thoracic contusions [116]. Two studies involving mouse PDGF‐responsive neural progenitors failed to improve behavioral function after rat contusion injury, indicating that neural transplantation doesn't always enhance neural function [103]. Nevertheless, the notion that Shiverer‐derived NPCs could impede endogenous cell remyelination and their inability to myelinate properly confounds those tests. A study explores the benefits of myelinating cell transplantation, identifying key substances released by these cells, and suggests systemic medications or protein infusions for remyelination and healing [120]. Transplanted cells can reduce inflammatory immune responses and promote anti‐inflammatory Treg signaling, potentially aiding clinical recovery in mouse models of demyelinating disease [121].

### 7.3 Hormones as a Target for OPC Differentiation and Remyelination Following SCI

After SCI, several hormones may help remyelination and improve functional recovery. Pregnancy can induce remyelination, a process that occurs after chemically induced demyelination, partly due to the upregulation of prolactin, a peptide hormone [86]. The thyroid hormone triiodothyronine (T3) is administered to enhance remyelination following cuprizone‐mediated demyelination [122]. It is also known that hormone receptors, such as the retinoid X receptor (RXR), contribute to remyelination. RXR belongs to the nuclear receptor family, and oligodendroglia produce more of this receptor during remyelination. To control various cellular functions, RXRs heterodimerize with liver X receptors, thyroid hormone receptors, vitamin D receptors, and peroxisome proliferation activator receptors [123]. After chemically induced demyelination, treatment with the RXR agonist 9‐cis retinoic acid is adequate to enhance remyelination and may be helpful after SCI [124]. Retinoids, which promote axonal regeneration after SCI, may aid in regenerating myelin and axons in the damaged spinal cord [125]. Myelin can impair brain function and lead to various neurological and mental disorders. Myelin protection and remyelination promotion are essential for various diseases. OLs and oligodendrogenesis produce myelin; many hormone classes regulate the lifelong process of producing new OLs. Hormone therapies offer potential ways to foster remyelination. Research on the hormonal control of oligodendrogenesis in diseases focuses on thyroid, peptide, and steroid hormones [126].

## 8 Myelin Loss and Oligodendrocytes: Preventing and Protecting

**FIGURE 4:**
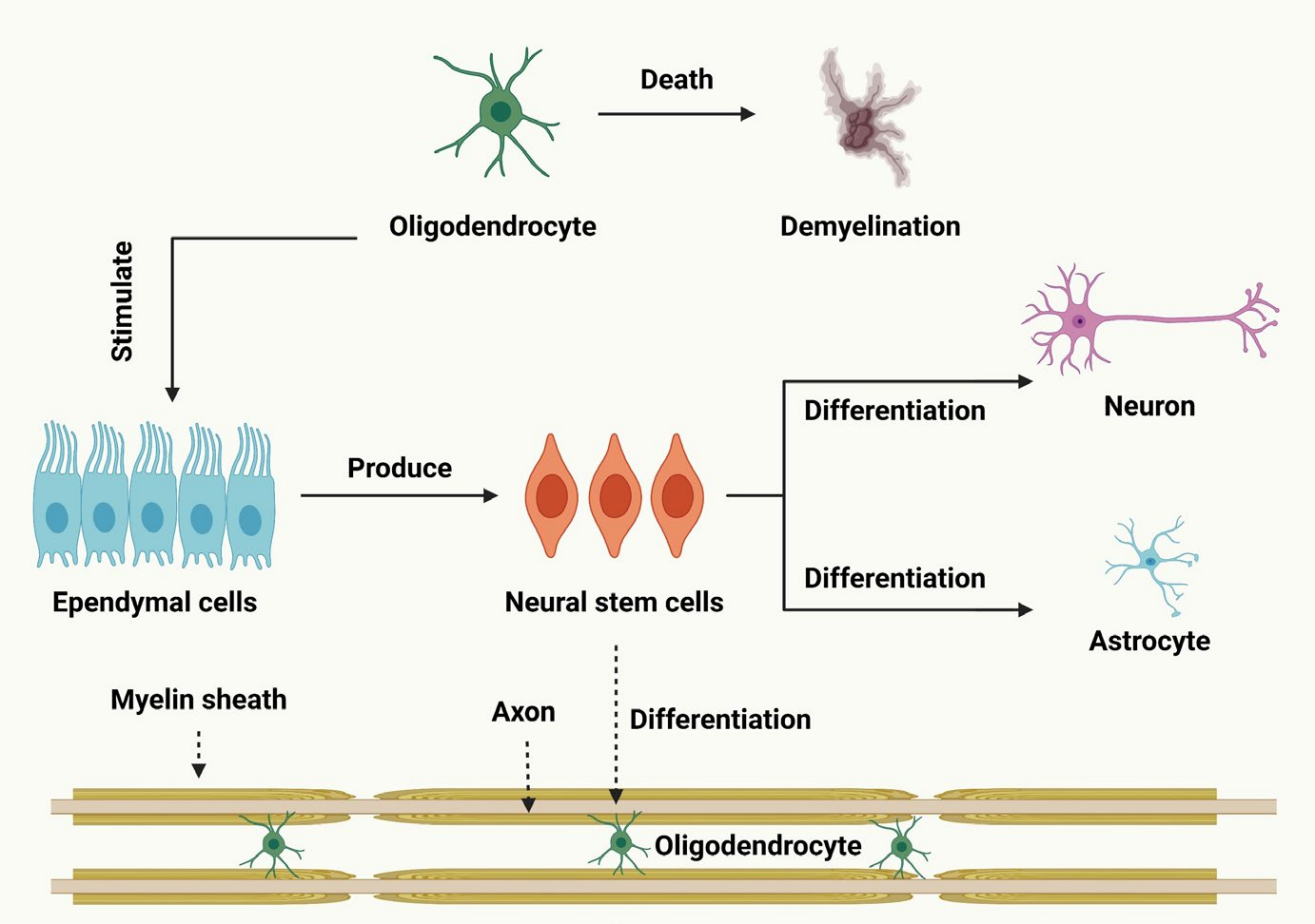
This process discusses demyelination and remyelination following SCI. SCI leads to demyelination, a loss of myelin sheath that impairs nerve function. Enhancing remyelination mechanisms could provide therapeutic intervention.

Myelin, a proteolipid sheath in the neurological system, helps with signal transduction. OLs, specialized glial cells, are responsible for myelin production and preservation. After SCI, oligodendroglia cell death and myelin degradation cause chronic axonal damage and loss of functions [127]. Several cellular and molecular processes lead to OLs (Figure 4) mortality during the acute and subacute secondary damage stages, ultimately resulting in myelin loss. OL necrosis or apoptosis is caused by a number of these processes, including hemorrhage, ischemia, the generation of free radicals, immune cell activation and infiltration, disruption of ion balance, and excitotoxicity [128]. In addition to being essential for axonal regeneration [129], preventing myelin degradation and/or removal of myelin debris is also necessary to differentiate OPCs into mature OLs during remyelination [105]. Stopping the self‐propelling mechanism of secondary damage after SCI is crucial as the OLs population around the lesion site has already decreased to half by day one. Given that the intervention time window is only open for the first 24 h, administering an extended halflife medication as soon as possible may be very beneficial clinically. It is essential to protect spinal interneuronal networks from harm before attempting to rebuild them because of their intricate structure and relationship with corticospinal motor neurons [130]. Early spinal decompression and stabilization surgery is increasingly popular for non‐life‐threatening SCI patients without medical comorbidities within 24 h of injury. It provides opportunities for local early‐phase drug delivery [131]. Myelinating glial cells, including OLs, are vital for brain function and computing by maintaining a balanced ionic environment, supplying metabolites, and determining conduction speed. The loss of this relationship contributes to neuropsychiatric diseases [132].

## 9 The Post‐SCI Remyelination Molecular Therapies

### 9.1 Guanosine

A promising therapeutic chemical in SCI, guanosine provides oligodendrocyte protection and can remyelinate. The chronic phase of SCI in rats, which began five weeks after the damage, was characterized by guanosine administration. After taking the medication continuously for seven days, they saw an improvement in locomotor function. Compared to the controls, there was an increase in mature oligodendrocytes and myelin production. It was proposed that the increased differentiation of migratory OPCs, aided by guanosine injection, was the cause of this increase in mature OLs [100]. The guanine accumulated in the spinal cord and stimulated the growth of NG2+ cells, including OPCs, which then matured into mature OLs [133].

### 9.2 Progesterone

Progesterone acts as a therapeutic agent in remyelination [134]. In comparison to controls, progesterone was found to increase remyelination in aged rats partially [135]. In rats with a transected spinal cord, it showed an increase in both OPC proliferation and remyelination [102]. The discovery of the intracellular progesterone receptor and membrane‐binding protein 25‐DX was founded on progesterone's effects on neurons and glial cells [136]. Progesterone enhanced BDNF expression, which in turn led to a notable rise in remyelination and an increase in OPC density at the site of damage [134].

### 9.3 Neurotrophins

Neurotrophic factors, including neurotrophin 3 (NT‐3) and PDGF, can stimulate OPC proliferation in vitro and influence their in vivo proliferation in stroke therapy [137]. It explores neurotrophic agents' impact on oligodendrocytes and OPCs, including neurotrophins (NT‐3, NGF, NT‐4, and BDNF) essential for CNS and PNS growth and maintenance [138]. Trks and p75NTR are key transmembrane receptors that interact with neurotrophins, with specific neurotrophins binding to specific Trk receptors in OLs from various species [139]. Researchers injected genetically engineered fibroblasts into animals with contused SCI to study neurotrophin application, finding BDNF and NT‐3 significantly improved remyelination post‐injection [101]. NT‐3 briefly impacts OPCs but significantly enhances myelination compared to BDNF and controls without proliferative effects on mature oligodendrocytes [140]. NT‐3 protects OPCs, promotes proliferation, differentiates into mature oligodendrocytes, increases susceptibility to necrosis, and has more TrkC receptors than mature oligodendrocytes. It has been demonstrated that NT‐3 treatment protects OPCs from glutamate excitotoxicity in three ways: (1) by increasing the number of OPCs to restore Ca^2+^ to normal; (2) by blocking a harmful downstream pathway brought on by elevated Ca2+ levels; or (3) by delivering its protective effects via a Ca^2+^‐independent pathway [141]. A study investigated the proliferative effect of NT‐3 on OPCs, demonstrating that it stimulates the transcription factor CREB [142]. CREB has been shown to enhance the anti‐apoptotic protein Bcl‐2 in OPCs, a promising factor for OPC survival post‐spine injury [143]. The virus infected GRP cells from 14‐day‐old embryos, causing normal myelin formation around axons and recovery in posterior limb locomotion in rats six weeks post‐implantation [144]. Transgenic Schwann cells expressing NT‐3 or BDNF showed higher functional recovery than the control group, primarily due to increased OPC differentiation and proliferation [145]. The administration of GDNF with Schwann cells and matrigel significantly improved neurite growth and remyelination compared to other treatments [146].

## 10 Remyelination via Cell Transplantation

Myelinating cell transplantation, a promising method for remyelinating damaged axons in SCI, is a promising alternative to losing myelin‐forming cells and demyelination [147]. Schwann cells, crucial for demyelinated axon regeneration after peripheral nerve damage, are genetically altered to improve their adaptability for CNS transplantation. In a mouse model, these cells showed faster functional recovery rates and improved locomotor performance after transplantation, highlighting their potential in CNS injury‐related remyelination therapy [148]. Olfactory unsheathing cell (OEC) transplantation offers superior axonal regeneration and widespread remyelination in human SCI, with early clinical trials indicating therapeutic potential [149, 150, 151]. A study on rats with transected spinal cords implanted OECs expressing GFP showed improved locomotor function and peripheral‐like myelinated axons derived from donor OECs [152]. Stem cell treatment for SCI in humans can be achieved through graft‐derived cell remyelination, demonstrating its biological significance for functional recovery in rats and monkeys [153].

## 11 Targeting Remyelination After SCI With Drug Delivery Systems

The effective drug delivery systems targeting remyelination, including MP, act as a potential therapeutic agent for acute SCI despite potential side effects like wound infections and pneumonia [154, 155]. A drug delivery system was developed using PLGA nanoparticles to encapsulate a quarter of the prescribed dosage and transport it to the injury site over time [156, 157]. MP‐encapsulated nanoparticles showed potent therapeutic effects compared to controls, resulting in smaller lesion locations, improved functional outcomes, and decreased marker responsiveness during secondary damage [157]. The hydrogel‐nanoparticle system was developed to transport MP to the spinal cord, allowing for less invasive and localized drug release. Seven days after SCI, MP diffused into the spinal cord, reducing activated microglia, proinflammatory protein expression, and lesion volume [156]. Chitosan nanospheres were investigated as potential drug delivery vectors that can cross the BBB to deliver caspase inhibitors [158, 159, 160]. The transferrin receptor, a widely distributed receptor in the brain capillary endothelium, was functionalized using PEG and an OX26 monoclonal antibody [159]. OX26 antibodies and chitosan nanospheres effectively target brain ischemia in mice, reducing caspase‐3 activity, neurological impairment, and infarction volume [160]. Thermosensitive agarose hydrogels release BDNF through lipid microtubules, facilitating neurite propagation and reducing reactive astrocytes and CSPGs [161]. A PEG‐based hydrogel with PLGA microparticles loaded with neurotrophins has been developed for targeting neuronal regeneration after SCI [162]. A new delivery method involves fibrin gel formation and immobilization of NT‐3 with heparin, resulting in increased neurite outgrowth and neural fiber density in SCI after nine days of implantation [163]. A nanomedicine‐loaded multitherapy, combined with ibuprofen and mouse nerve growth factor, has shown short‐term anti‐inflammatory effects and long‐term improvements in myelin regeneration and SCI prognosis [13].

## 12 Conclusions and Future Perspectives

The study significantly enhances our understanding of remyelination mechanisms after SCI and emphasizes the therapeutic potential of targeted interventions for improved myelin repair. We demonstrated the possibility of stimulating OPC proliferation, differentiation, and myelin sheath formation in both in vitro and in vivo SCI models through molecular pathway modulation and pharmacological agents. The observed improvements in remyelination demonstrate the effectiveness of targeted therapies in promoting neurological recovery after SCI. The transition from experimental success to clinical application is a complex process requiring thorough safety evaluations, system optimization, and the identification of optimal therapeutic windows. Extensive preclinical studies are needed to understand optimal conditions for effective therapies, including dosing regimens and treatment timing. The development of non‐invasive imaging techniques for real‐time monitoring of remyelination is crucial for evaluating treatment efficacy in clinical trials. The increasing complexity of SCI, leading to increased effects on the CNS, requires combinatory therapies targeting neuroinflammation, axonal degeneration, and remyelination. These approaches may provide complementary advantages, potentially enhancing the overall recovery outcomes. Utilizing genomic and proteomic profiling, personalized medicine could significantly optimize SCI treatment strategies by providing therapeutic interventions. Advanced stem cell therapy techniques and targeted remyelination therapies could significantly improve the treatment of SCI by replacing damaged cells and developing neuronal regeneration and functional recovery. The study provides a foundation for developing targeted remyelination therapies as a promising avenue for SCI treatment.

## Author Contributions

**Abdullah Al Mamun:** conceptualization, visualization, writing – original draft; writing – review and editing. **Zhou Quan:** conceptualization, visualization, writing – original draft, writing – review and editing. **Peiwu Geng** and **Shuanghu Wang:** conceptualization, visualization, writing – original draft, writing – review and editing. **Chuxiao Shao and Jian Xiao:** conceptualization, funding acquisition, project administration, resources, supervision, validation, writing – original draft, writing – review and editing.

## Conflicts of Interest

The authors declare no conflicts of interest.

## Data Availability

The data that support the findings of this study are available from the corresponding author upon reasonable request.
